# On the past, present and future of senotherapeutics

**DOI:** 10.1038/s41514-024-00139-3

**Published:** 2024-02-03

**Authors:** Marco Quarta, Marco Demaria

**Affiliations:** 1 Rubedo Life Sciences, Sunnyvale, CA USA; 2 European Research Institute for the Biology of ageing (ERIBA), University Medical Center Groningen (UMCG), University of Groningen (RUG), Groningen, Netherlands

**Keywords:** Senescence, Translational research

During the first senotherapeutics conference organized by the Phaedon Institute at the Buck Institute for Research on Aging (Novato, CA), experts on the molecular and cellular mechanisms of aging, pathogenesis of age-related diseases, and drug discovery and development convened to delve into ideas on the past, present, and future of targeting senescent cells. The summit was divided into four sessions: basic biology, preclinical, clinical, and early-stage companies. Panel discussions covering large pharma, biomarkers, and investments in the field were organized between sessions.

A focal point of discussion revolved around the heterogeneity of cellular senescence, and its profound implications for the development of treatments. Presenters highlighted the diverse profiles of senescent cells, emphasizing differences in gene expression, secretory patterns, and functional roles, in addition to the importance of the tissue microenvironment.

While senescent cells are viewed as a major contributor to aging-associated dysfunctions, the conference highlighted the nuanced roles they play. Some subsets of senescent cells contribute to tissue repair and regeneration; thus, their elimination could potentially lead to toxicity. Senescent-like terminally differentiated or post-mitotic cells, such as neurons, adipocytes, and cardiomyocytes, maintain partial functionality when expressing senescence markers, raising questions on whether their elimination could cause harm. Similarly, macrophages tend to express several senescence markers, even when their function is not compromised. Compelling evidence was provided for the co-existence of subsets of senescent cells uniquely expressing p21 or p16, both characterized by unique functional and secretory profiles and present in tissues at various life stages.

Understanding the phenotypic diversity of various subsets, and the pathophysiological contexts in which they are implicated, is crucial for developing precise therapeutic strategies that target specific senescent cell populations. Researchers have presented novel senolytic approaches that target senescence-associated survival mechanisms. The potential of modulating the senescence-associated secretory phenotype (SASP) to steer senescent cells towards a more regenerative phenotype has also emerged as a promising avenue for therapeutic intervention. In addition, the use of surface proteins for immune-mediated clearance was discussed.

Senescent cells represent a potential target for geroprotection and reduction of multimorbidity, but owing to current regulations, clinicians and pharmaceutical companies are focusing on the use of senotherapeutics for specific and selective age-related diseases. A Phase 2 B trial sponsored by Unity Biotechnology is currently ongoing for the treatment of Diabetic Macular Edema (DME) using the senolytic agent UBX1325, which inhibits Bcl-XL. The Translational Geroscience Network runs a number of Phase 1 and Phase 2 trials using senolytic compounds, such as Dasatinib, Quercetin, and Fisetin, for the treatment of sepsis, chronic kidney disease, lung fibrosis, and Alzheimer’s disease. Additionally, many other pathological conditions have been discussed as potential indications for the use of therapeutic compounds. For example, efforts are currently being made to selectively target senescent cell subtypes in pre-clinical models of skin and muscle dysfunctions with novel senolytic small molecules in development at Rubedo Life Sciences and Boehringer-Ingelheim, respectively.

While these represent pioneering studies and the opportunity to demonstrate the unequivocal pathological role of certain senescence subsets, challenges remain in the path to harnessing the therapeutic potential of targeting cellular senescence. Participants emphasized the need for a more comprehensive understanding of the dynamic nature of senescent cells, their role and localization in various tissues and tissue areas, and the need for more accurate and sensitive biomarkers. This discussion highlighted the importance of multi-laboratory and multi-center efforts in adapting the newest technologies with single-cell resolution for the identification and specification of senescent cells in vivo. These studies can offer novel targets for interventions and novel markers for a standardized evaluation of the efficacy of anti-senescence approaches in humans. Overall, the pleiotropic diversity and heterogeneity of cellular senescence invites the development of diverse strategies and modalities to target subsets of senescent cells according to their physiopathological roles. The ongoing effort in academic and industrial laboratories to develop different senotherapeutics is strategic to enable advancement in the clinic of multiple novel therapeutic opportunities, possibly in parallel.

In conclusion, the summit underscored the importance of embracing diversity in senescent cell populations, and highlighted the potential for developing targeted treatments against age-related diseases. In the coming years, it will be important to stimulate more investments in the field from governmental and non-profit agencies, but also from venture firms and large pharmaceutical companies. As research in this field continues to evolve, more funding will facilitate a deeper understanding of the phenotypes and functions of cellular senescence in vivo*,* and bring us closer to innovative therapies that could redefine how we approach aging and its associated dysfunctions.

